# Rapidly growing primary anal canal lymphoma: a case report and literature review

**DOI:** 10.1093/gastro/goad058

**Published:** 2023-09-22

**Authors:** Yu Kyung Chung, Su Bum Park

**Affiliations:** Department of Internal Medicine, Pusan National University School of Medicine and Research Institute for Convergence of Biomedical Science and Technology, Pusan National University Yangsan Hospital, Yangsan, Republic of Korea; Department of Internal Medicine, Pusan National University School of Medicine and Research Institute for Convergence of Biomedical Science and Technology, Pusan National University Yangsan Hospital, Yangsan, Republic of Korea

## Introduction

Anal cancer is a rare cancer accounting for ∼2% of all gastrointestinal tract malignancies [1]. Among anal cancers, squamous cell cancer (SCC) is the most common and lymphoma is one of the least frequent. The gastrointestinal tract is the most common site for extranodal non-Hodgkin’s lymphoma (NHL), and diffuse large B-cell lymphoma (DLBCL) is the most common histologic subtype. However, primary colorectal lymphoma accounts for only 0.3% of all large intestinal malignancies and ∼3% of all gastrointestinal lymphomas [2]. Anal canal SCC and lymphomas are treated differently; thus, accurate diagnosis is crucial.

Herein, we present the case of a 63-year-old man who was diagnosed with primary anal canal lymphoma that was completely resolved after six cycles of chemotherapy. This report focuses on anal canal lymphoma and reviews the relevant literature.

## Case report

A 63-year-old man presented having had anal pain and hematochezia for 1 month. He also reported frequent defecation (five times/day), tenesmus, and weight loss of 4 kg during the same period. The patient had no relevant medical history apart from dyslipidemia and no significant surgical, social, or family history. Physical examination findings were normal except for an anorectal mass palpable on digital rectal examination. No superficial lymph nodes (LNs) were palpable.

**Figure 1.**
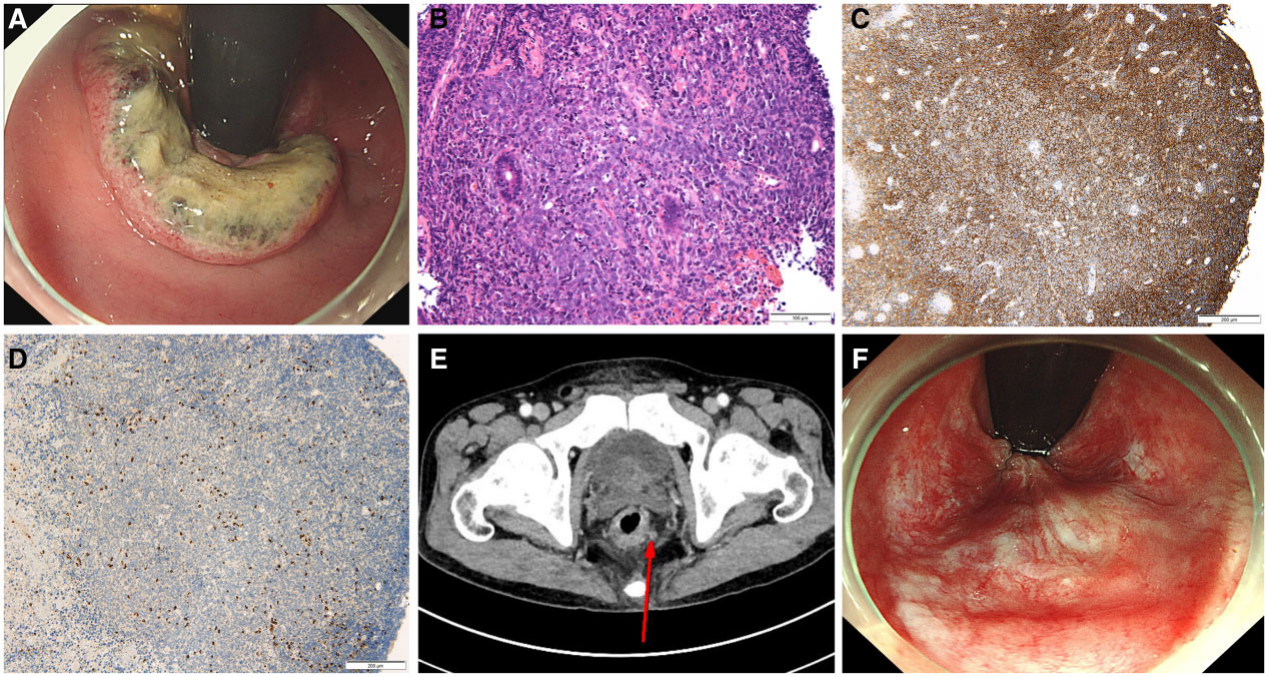
Primary anal canal lymphoma successfully treated with R-CHOP chemotherapy. (A) An ulcerative fungating mass covered with dirty exudate on the rectum and anus, which was observed at diagnosis. (B) Hematoxylin and eosin staining (×200); photomicrograph shows a large atypical pleomorphic lymphocytic nucleus with multiple nucleoli. (C) CD20 immunostaining positive (×100). (D) CD3 immunostaining negative (×100). (E) Computed tomography shows a mass encircling the rectum with mesorectal lymph node (arrow). (F) Whitish scars on the rectum and anus, which were observed 6 months after treatment. R-CHOP, rituximab, cyclophosphamide, doxorubicin, vincristine, and prednisolone.

Laboratory tests revealed mild anemia (hemoglobin, 12.2 g/dL) and other routine hematological and biochemical investigations, including tumor markers (carcinoembryonic antigen and carbohydrate antigen 19–9) and lactate dehydrogenase, were within the normal range. Human immunodeficiency virus antibody test and Epstein-Barr virus (EBV) DNA results were negative. Colonoscopy revealed an ulcerative fungating mass with a soft margin that extended from the anal rim to the rectum, measuring ∼5 cm on the anal canal (Figure 1A). Histologic examination of the biopsy samples revealed a diffuse proliferation of large atypical lymphoid cells with a high nuclear–cytoplasmic ratio, coarse chromatin, and prominent nucleoli (Figure 1B). The immunohistochemical staining results were as follows: positive for CD20 (Figure 1C), BCL2, MUM1, and c-MYC (moderate intensity, ∼50%) and negative for CD3 (Figure 1D), CD10, and BCL6. The fluorescence *in situ* hybridization results for MYC and EBV with *in situ* hybridization were negative. Chest computed tomography (CT) revealed no evidence of lymphoma, and enhanced abdominal/pelvic CT and magnetic resonance imaging showed a mass encircling the rectum and enlarged regional LNs (Figure 1E). Positron emission tomography–CT revealed no malignant tumors other than the anal mass. Bone marrow biopsy showed no definite evidence of the involvement of the malignant lymphoma. The tumor was located only in the anal canal, with regional LN spread.

Based on the clinical, histopathologic, and immunohistologic features, the patient was diagnosed with primary anal canal DLBCL and regional LN involvement (stage IIE 1, international prognostic index of 1). A rituximab, cyclophosphamide, doxorubicin, vincristine, and prednisolone (R-CHOP) chemotherapy regimen was performed for six cycles. Subsequent CT and colonoscopy with a biopsy of the lesion revealed that the mass had completely disappeared (Figure 1F). The patient was doing well without any evidence of recurrence at the last follow-up and continues to be followed up regularly.

## Discussion and conclusions

Cancers of the anal canal represent ∼2.4% of all gastrointestinal neoplasms. Anal lymphoma represents 0.2% of anorectal tumors, most of which correspond to NHLs [3]. The common site for gastrointestinal lymphoma is the cecum, which is involved most commonly (60%–70%), followed by the right colon [4]. Primary anal canal NHL is a very rare disease.

The symptoms of gastrointestinal lymphoma vary, including abdominal pain, weight loss, mass, and hematochezia, in addition to the features of obstruction, such as nausea, vomiting, and changes in bowel habits [5]. In our patient, the sites involved were the rectum and anus; thus, symptoms such as hematochezia and anal discomfort could lead to an early diagnosis. The most frequent symptoms include the sensation of a perianal mass, chronic ulceration, and bleeding in the anal canal lymphoma. The last colonoscopy for this patient, which was performed 5 months before the diagnosis, showed no lesions in the rectum and anus, indicating that the tumor had grown rapidly during the intervening 5 months. Considering the speed of the tumor growth, had there been no symptoms, the disease could have progressed to a more advanced stage.

The clinical presentation of anal canal lymphoma usually mimics those of other anal cancers, such as anal SCC. Lymphomas are malignant neoplasms of lymphocyte cell lines that originate from the deep mucosa or submucosa; therefore, lymphomas can appear as sub-epithelial lesions. In this case, colonoscopy revealed a mass with central ulceration and a soft margin, which could be seen in the sub-epithelial lesion.

In these cases, colonoscopy with biopsy is the most useful diagnostic test. Obtaining adequate samples that allow histopathological diagnosis is vital for establishing appropriate treatment. If lymphoma is suspected based on the initial hematoxylin–eosin staining, immunohistochemical assessment is conducted on fixed, paraffin-embedded tissue specimens using antibodies specific for differential diagnosis. Chest and abdominal/pelvic CT is recommended, and bone marrow biopsy and positron emission tomography are recommended for proper staging. In our patient, the final diagnosis was primary DLBCL of the anal canal with Ann Arbor stage IIE 1, which is defined as a tumor with regional LN involvement and an international prognostic index of 1.

The treatment for anal canal lymphomas remains uncertain. While surgical treatment may be indicated for some localized tumors, medical management is considered the primary treatment [6]. Rapidly proliferating and aggressive advanced lymphomas are best treated with chemotherapy. The addition of rituximab to the standard CHOP chemotherapeutic regimen has led to improvements in progression-, event-, and disease-free and overall survival [7]. In this case, the tumor was successfully treated with R-CHOP. Radiotherapy in anal lymphomas can be complementary, especially in local recurrences or in patients who have undergone local resections with positive margins [8].

In conclusion, malignant anal canal lymphomas are not easy to distinguish from other anal cancers based on endoscopic features without histopathologic examination. However, when lesions with a relatively soft margin exhibit a rapid growth pattern, the occurrence of primary anal canal lymphomas is possible. Accurate diagnosis using a multimodality approach and proper treatment can improve the patient’s prognosis.

## Authors’ Contributions

S.B.P. and Y.K.C.: acquisition of data, drafting of the manuscript, critical revision of the manuscript for important intellectual content, and study supervision. All authors read and approved the final manuscript.
